# How far *in-silico *computing meets real experiments. A study on the structure and dynamics of spin labeled vinculin tail protein by molecular dynamics simulations and EPR spectroscopy

**DOI:** 10.1186/1471-2164-14-S2-S4

**Published:** 2013-02-15

**Authors:** MNV Prasad Gajula, KP Vogel, Anil Rai, Franziska Dietrich, HJ Steinhoff

**Affiliations:** 1 CABin division, DST Ramanujan Fellow, Indian Agricultural Statistics Research Institute, PUSA campus, New Delhi-110012, India; 2 Department of Physics, University of Osnabrueck, Barbara strasse-7, D49076-Osnabrueck, Germany; 3 IZKF Leipzig, Faculty of Medicine, University of Leipzig, Liebigstr. 21, D-04103 Leipzig, Germany

## Abstract

**Background:**

Investigation of conformational changes in a protein is a prerequisite to understand its biological function. To explore these conformational changes in proteins we developed a strategy with the combination of molecular dynamics (MD) simulations and electron paramagnetic resonance (EPR) spectroscopy. The major goal of this work is to investigate how far computer simulations can meet the experiments.

**Methods:**

Vinculin tail protein is chosen as a model system as conformational changes within the vinculin protein are believed to be important for its biological function at the sites of cell adhesion. MD simulations were performed on vinculin tail protein both *in water *and *in vacuo *environments. EPR experimental data is compared with those of the simulated data for corresponding spin label positions.

**Results:**

The calculated EPR spectra from MD simulations trajectories of selected spin labelled positions are comparable to experimental EPR spectra. The results show that the information contained in the spin label mobility provides a powerful means of mapping protein folds and their conformational changes.

**Conclusions:**

The results suggest the localization of dynamic and flexible regions of the vinculin tail protein. This study shows MD simulations can be used as a complementary tool to interpret experimental EPR data.

## Background

Rapid advances in computer technology have led to the development of successful molecular simulations of protein structural dynamics that are intrinsic to understand biological processes. These simulations have resulted in the development of novel models and methods that increasingly agree with experimental observations, and suggest new experiments providing insights into biological mechanisms. Used in combination with the information gained by sophisticated experimental techniques, molecular simulations can help us, to understand biological complexity at the atomic and molecular levels. Here, we emphasize such an approach that illustrates the potential of molecular dynamics simulations in analyzing experimental results determined by EPR spectroscopy on vinculin as an example.

**Figure 1:**
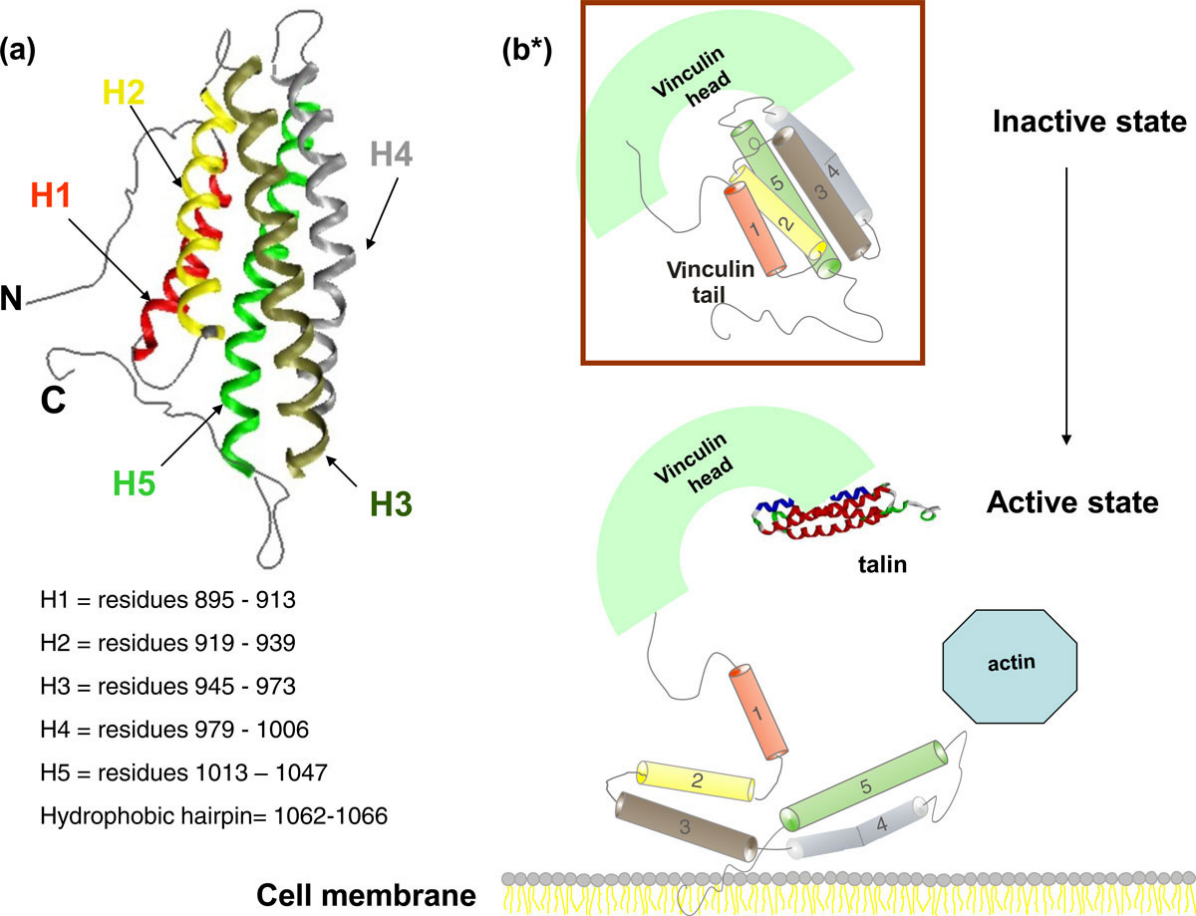
**Schematic representation of structure and function of vinculine tail**. (a) Vinculin tail domain with its 5 helix pack; Each helix is shown in different color for easy identification. N and C-terminal domains are shown as threads. The residue numbers of each helix are given. (b) Schematic representation of the functional domains of vinculin and some of its ligands. Vinculin is supposed to cycle between active and inactive conformations. Insertion of the hydrophobic hairpin(C-terminal) into the membrane is thought to be the primary step involved in the Vt detachment from its head. (b)(top) Inactive state of the vinculin is shown in box. In this state, vinculin is held in a "closed", auto-inhibited conformation by intra-molecular interactions between the head and tail domains. (b)(bottom) A model of vinculin activation. Conformational changes in the C-terminal tail domain are thought to play a key role in this action, but there are no significant studies so far that provide the active state conformation in living cells. *Inspired from [5,6]. For more extensive reviews also refer to [3,28].

Vinculin is a highly conserved intracellular protein that plays a critical role in cellular adhesion, migration, maintenance and regulation of cell shape [1-3]. Vinculin exists in two distinct conformations depending on an intra-molecular interaction between its head (Vh) and tail (Vt) domains (cf. Figure 1) [4-6]. Conformational changes within the vinculin protein are believed to be important for its biological function [7-9]. Despite intensive biophysical and biochemical studies, the dynamics of vinculin activation are still unclear [10-14]. Conformational changes in the C-terminal tail domain are thought to play a key role in this action. Here, we employ molecular dynamics simulations to study the site specific behaviour of the vinculin tail in its inactive conformation. We focus on the dynamics of spin labels that are bound to specific protein regions and which could provide insight into possible conformational changes during protein function.

Site directed spin labeling EPR spectroscopy has evolved as a powerful technique to investigate protein structure and conformational changes under physiological or near-physiological conditions [15-18]. The shape of continuous wave (cw) EPR spectra recorded at room temperature is sensitive to the re-orientational motion of the bound spin label side chain providing information on the motional restriction of the nitroxide due to sterical interaction with the secondary and tertiary structure [18-20]. In addition, solvent accessibility of the spin label side chain and polarity of the nitroxide microenvironment characterize the protein topology. Dipolar coupling between two spin labels incorporated into a protein report on intra-molecular distances [21,23]. Details of this method are summarized in recent reviews by Bordgnon et al, and Klare et al. Here we report on MD simulations of spin labelled vinculin tail in order to analyze experimental spin label EPR spectra.

## Methods

The initial coordinates for the vinculin tail MD simulations were obtained from the Protein Data Bank (PDB code: 1ST6). All MD simulations were performed with the GROMACS simulation suite for *in water *and *in vacuo *simulations. The force field ffG43a1 was used for the simulations *in water *and the force field ffG43b1 from GROMOS, which is integrated into GROMACS, was used for the simulations *in vacuo*. Several MD simulations were performed with different spin labeled sites for 30 ns at 300 K in water, whereas the simulations *in vacuo *were performed for 10 ns at 600 K. In order to assure that the spin label covers the maximum accessible conformational space within a relative short MD run of 10 ns, a high temperature of 600 K was applied with position restraints on all backbone atoms. Periodic boundary conditions were applied as mentioned elsewhere. For a detailed description of the MD simulations methodology and applications refer [25,29,30]. The vinculin tail protein holds two native cysteines at positions 950 and 972 on helix 3. Cysteines were genetically introduced at positions 901, 909 on helix 1 (H1); 922,927,934 on helix 2(H2); 957 on helix 3(H3); 984 on helix 4(H4); 1024, 1033 on helix 5(H5); and 1062 in the C-terminus. EPR experiments on these mutants were described in [20]. Refer to [24-26] for calculating EPR spectra from MD simulations data.

## Results

### Spin label dynamics *in water *and *in vacuo*

**Figure 2:**
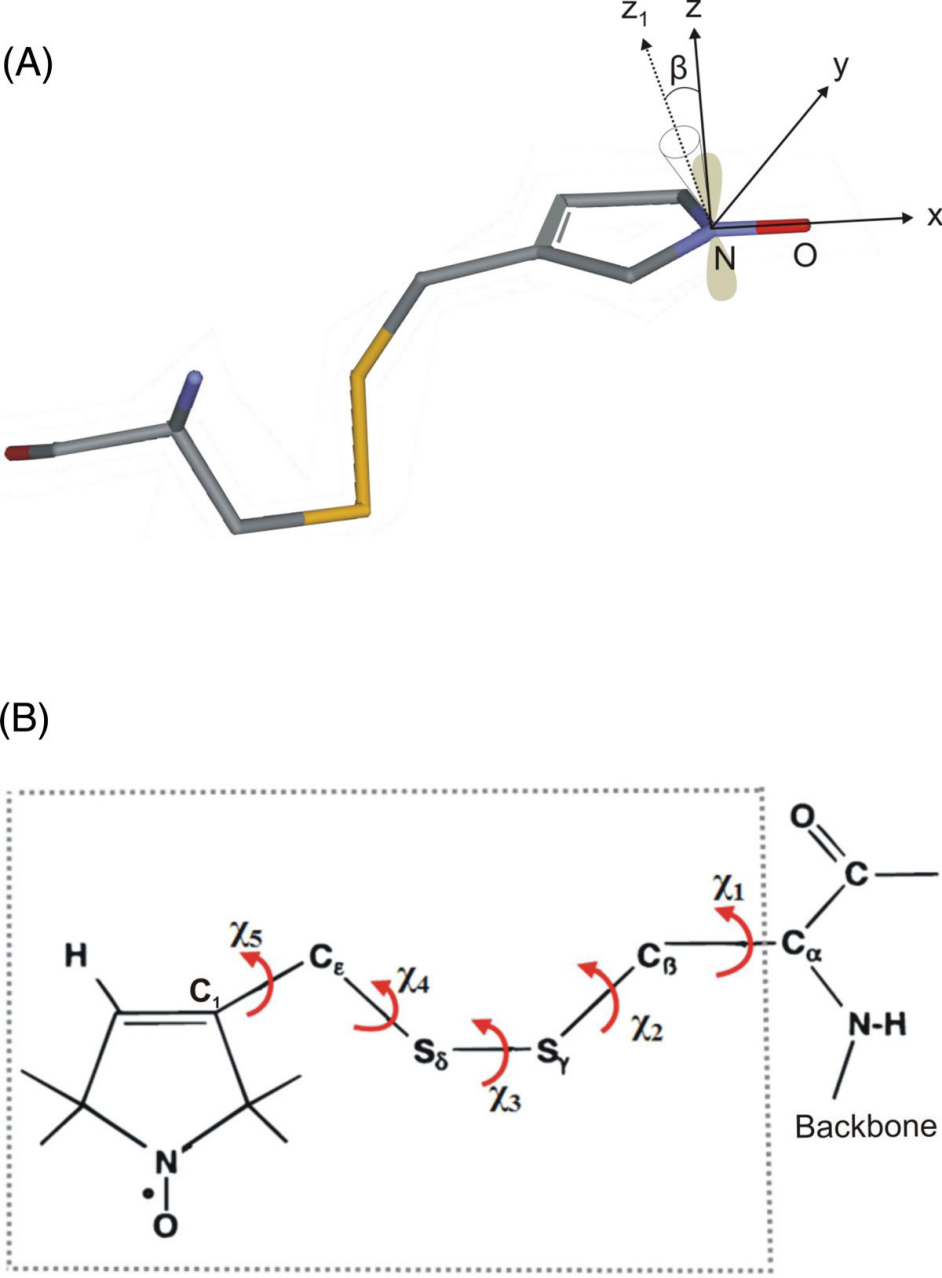
**Spin label side chain**. (a) Cysteine bound spin label MTSSL. The molecular coordinate system (x, y, z) of a nitroxide with x along the N-O bond and z perpendicular to the plane of the nitroxide for z-axis anisotropic motion is depicted. The orientation of the tether with respect to the nitroxide axis system is determined by the angles α, β and γ (α and γ not shown). β is the angular deviation from the mode of the orientational distribution of the z-axis of the nitroxide. The nitrogen *p *orbital along the z-axis is shown in shaded color. For simplicity, the methyl substituents of the nitroxide ring are not shown. (b) All flexible bonds within the spin label side chain (shown in dotted box). The interaction between the Sγ and Cα proton limits the rotmers Cα-Cß, Cß-Sγ, Sγ-Sδ.

**Figure 3:**
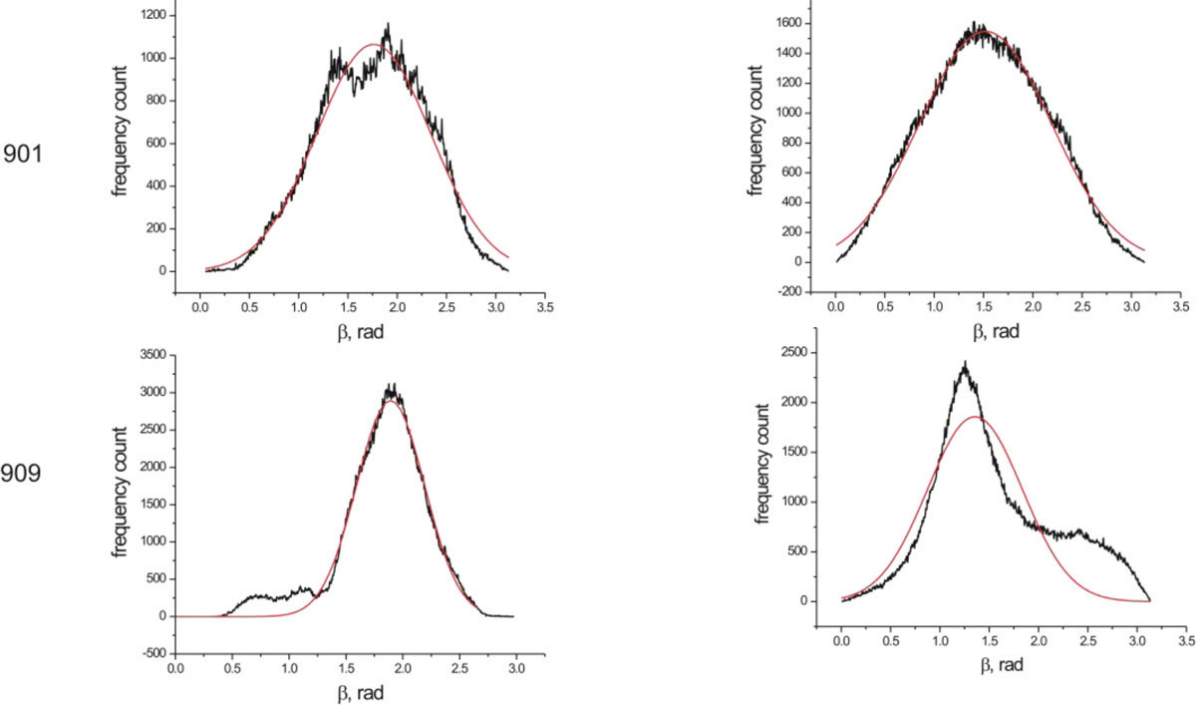
**Distribution of angle β**. Distribution of angle β determined from MD simulations in water (left) and in vacuo (right). Residue numbers are given in front of the respective plot. (Gaussian fittings are shown in red).

**Figure 4:**
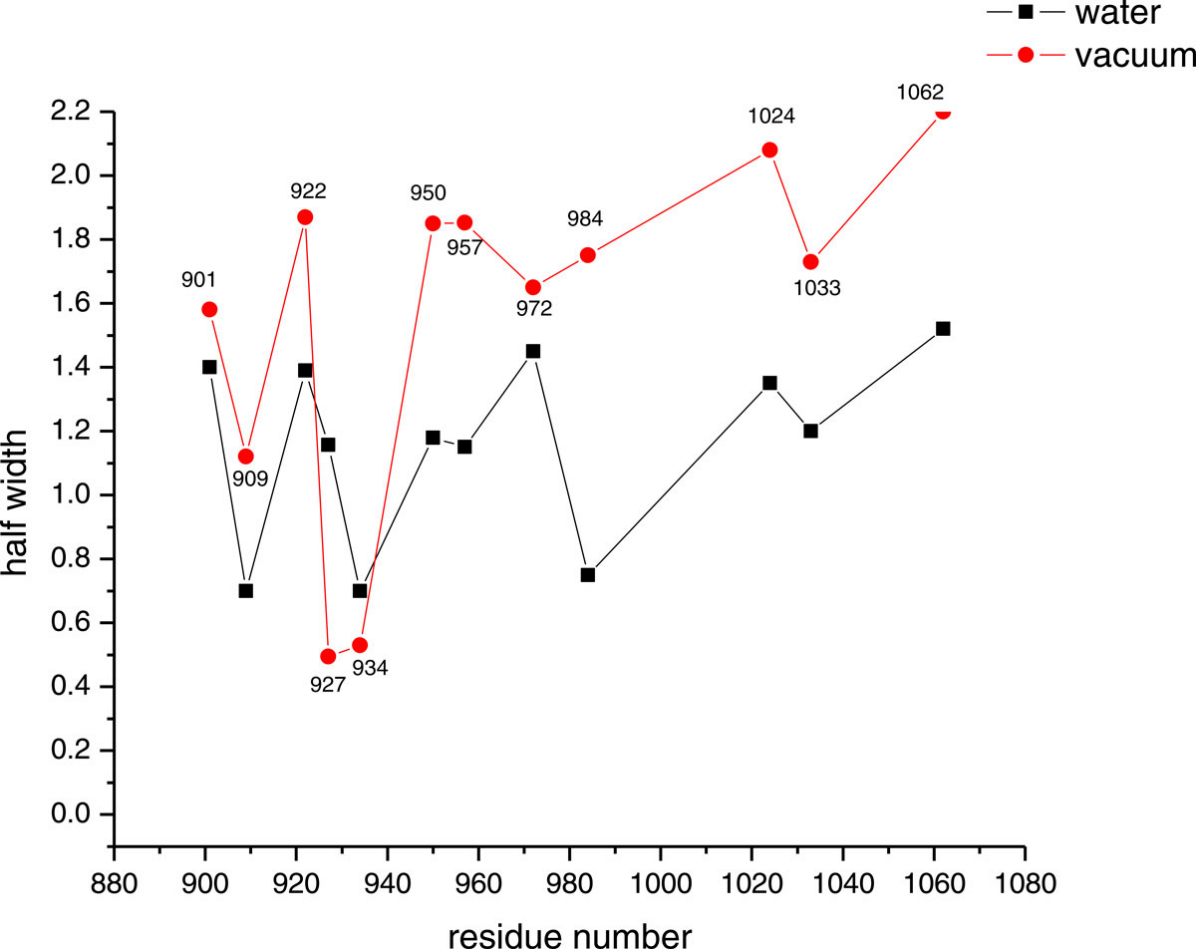
**Comparison of distribution of angle β**. Comparison of the full width at half height values of the angle β distributions of the nitroxide side chains both *in water *and *in vacuo *simulations.

MD simulations were performed in water and in vacuo environments as described in the methods section on a series of selected spin labeled sites specifically bound to the vinculin tail protein. In order to characterize the dynamics of the spin-labeled side chains, the reorientational dynamics are represented by the mean fluctuation of the nitroxide ring orientation as shown in Figure 2. Here the angle β is the angular deviation from the mode of the orientational distribution of the z- axis of the nitroxide spin label. (see [24-26] for more information about the angle β). As an example, the distributions of β for only two spin labeled residues, positions 901 and 909 are shown in Figure 3, the widths of the distributions for all samples are depicted in Figure 4. The β distribution widths determined from MD simulations in vacuo and in water show similar behaviour. In both cases the calculations reflect the dependency of the spin label mobility on the specific location.

### Mobility parameter

**Figure 5:**
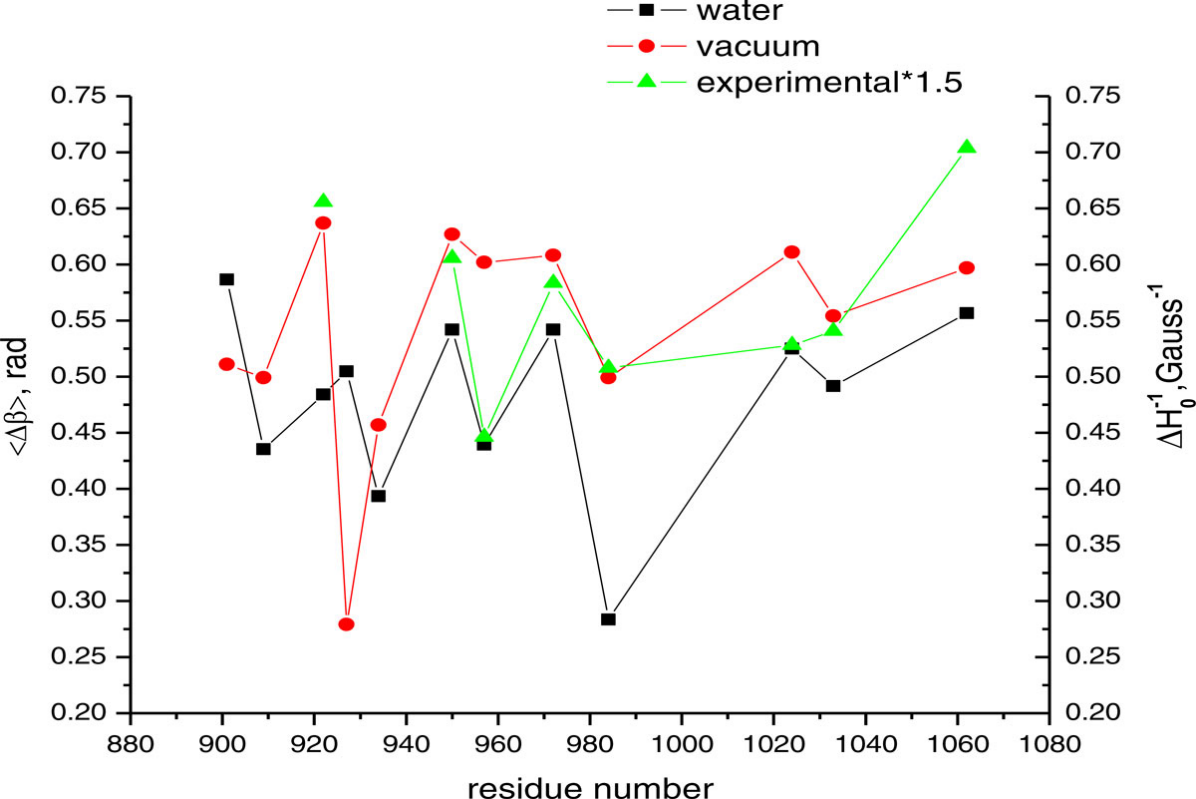
**Measures of mobility.** Measures of mobility for twelve representative nitroxide side chains at various positions in the vinculin tail protein.

For a quantitative comparison of experimental and simulated data we calculated mobility parameters [26,27]. The mean square fluctuation amplitude of the nitroxide (〈*Δβ*〉)^2 ^is determined from the simulations. The inverse line width of the central resonance $\Delta {\text{H}}_{0}^{-1}$ of EPR spectra is a convenient experimental measure of the nitroxide mobility that can be compared to the MD simulations results. The higher these mobility parameters, the higher the flexibility of the spin label side chain. The nitroxide mobility from the series of vinculin tail variants discussed above is projected in Figure 5 as function of residue number. The experimental values were taken from [20].

### Comparison of simulated and experimental data

**Figure 6:**
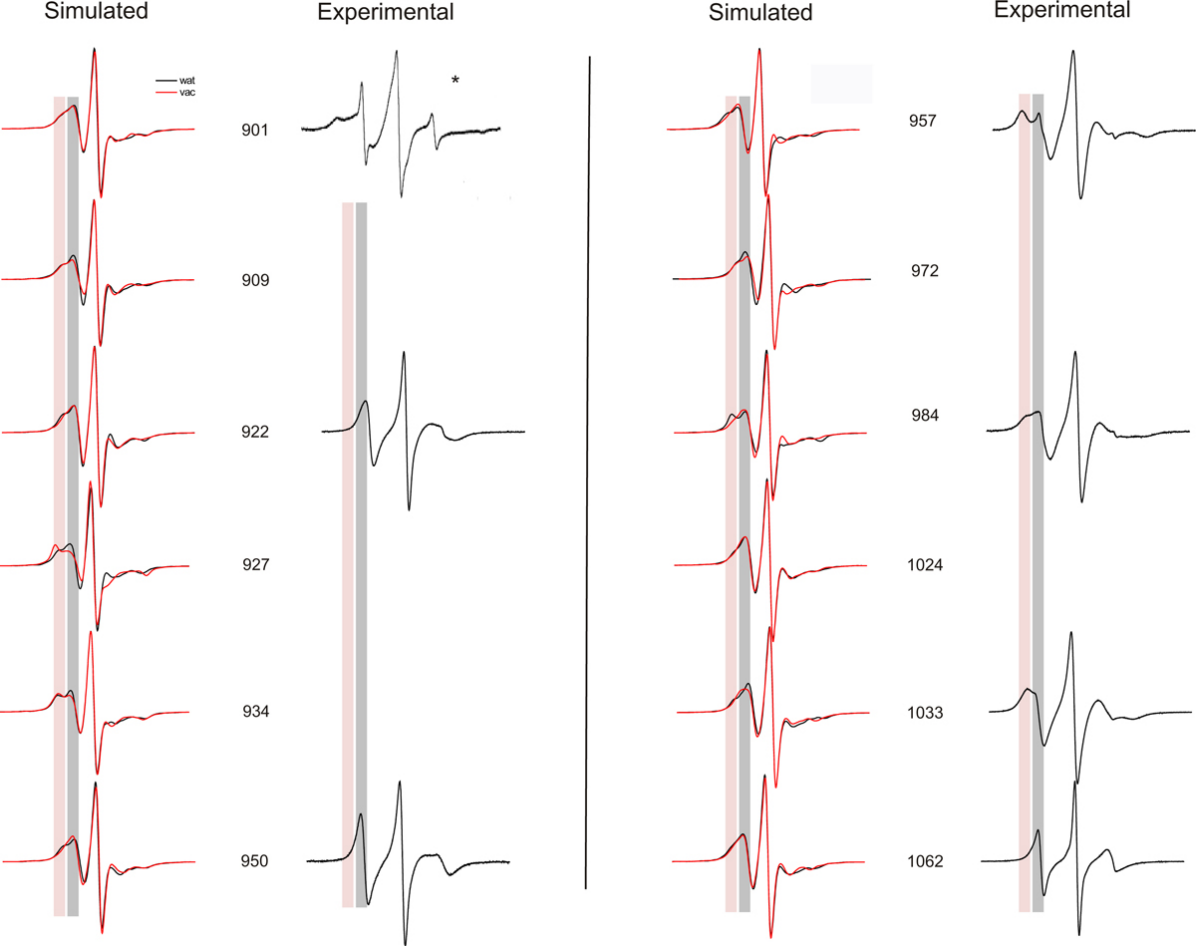
**Simulated and experimental EPR spectra**. Simulated and experimental EPR spectra for various positions in the vinculin tail domain. Residue positions of the spin labeled sites are indicated. EPR experimental data is not available for positions 909, 927, 934, 972 and 1024. Simulated EPR spectra were calculated based on MD simulations in vacuo (red) and in aquo (black). Red and gray colored bars mark spectral region indicative for an immobile or mobile spin label side chain. * courtesy: Christoph Abe and Wolfgang Ziegler.

In addition to the reorientation angle analysis, EPR spectra were calculated from MD simulations data according to the method described in [24-27]. The simulated spectra from MD data were compared with those of experimental EPR spectra of spin labels attached to various positions of the vinculin tail that are presented in Figure 6. Most of the spectra show two distinct components that reflect the different dynamic modes of the spin label side chain depending on the interactions with nearby residues and helical contacts. In Figure 6, immobile and mobile componants are indicated by the red and gray bar respectively. In addition, a high mobility of the spin label may indicate a flexible region of the protein. The simulated spectra of spin labels at positions 901 to 922 show similar behaviour both *in water *and *in vacuo *simulations. The β distributions (Figures 3 and 4) support this data. An experimental spectrum is available for position 922 that reveals a higher mobility compared to the simulated spectra. A different mobility pattern is seen *in water *and *in vacuo *simulations for the positions 927 and 934. *In vacuo *simulations show a decrease in the mobility for both positions reflecting more backbone or tertiary interactions. Unfortunately, experimental spectra are not available for these two positions. 950R1 shows a similar behaviour both *in water *and *in vacuo *simulations. Again the experimental data reveal higher mobility. In contrast, the simulated spectra of the spin label at 957 show higher mobility than revealed in the experimental spectrum. Water simulations of the spin labels attached to 984 in helix 2 show similar behavior to the experimental spectrum, whereas the spectra determined from MD simulations performed *in vacuo *differ with that data. The spin label at position 1024 shows an increased mobility in both simulated and experimental spectra. A small immobile peak in the experimental spectrum suggests a significant interaction for this position with the surrounding residues. The simulated spectrum for 1033R1 in vacuo is well in agreement with that of the experimental spectrum with a decrease in mobility. Again the spin label at position 1062 shows similar behavior in both simulated and experimental spectra, with the experimental spectrum revealing less motional restrictions. When compared to the experimental data, the dynamics of the spin labels at positions 957, 1024 and 1033 show higher mobility in simulations, whereas the tendency is true for 922, 950, and 1062.

### Inter helical distances

**Figure 7:**
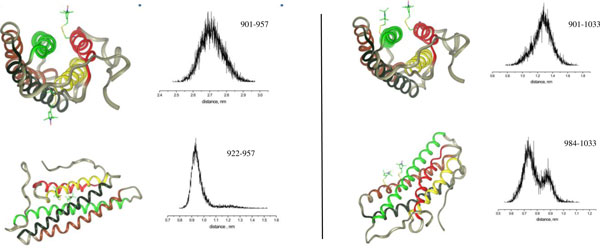
**Inter helical distances.** Vinculin tail domain with bound spin labels (left) and corresponding interspin distance distributions (right).

Inter helical distance measurements within the vinculin tail are good means of representing the tertiary structure. In addition, they give a highly localized view of the conformational dynamics of the polypeptide chains. The results presented in Figure 7 show the distances between selected spin labeled sites within the vinculin tail domain as simulated by in vacuo MD simulation. Sites were chosen according to available experimental data [20]. Experimental and simulated inter residue and inter nitroxide distances are listed in Table 1.

**Table 1:** Distance comparisons

Residues	X-ray (Cα-Cα), Å	MD (NX-NX), Å	EPR (NX-NX), Å
901-957	16.4	27.2	26.5 ± 1
922-957	5.53	9.5	8 ± 1
901-1033	11.37	12.7	8 ± 1
984-1033	6.58	7.3(~65%) 9(~35%)	7 ± 1

Comparison of distances from X-ray structure, MD simulations and EPR experiments [20]

## Discussion

**Figure 8:**
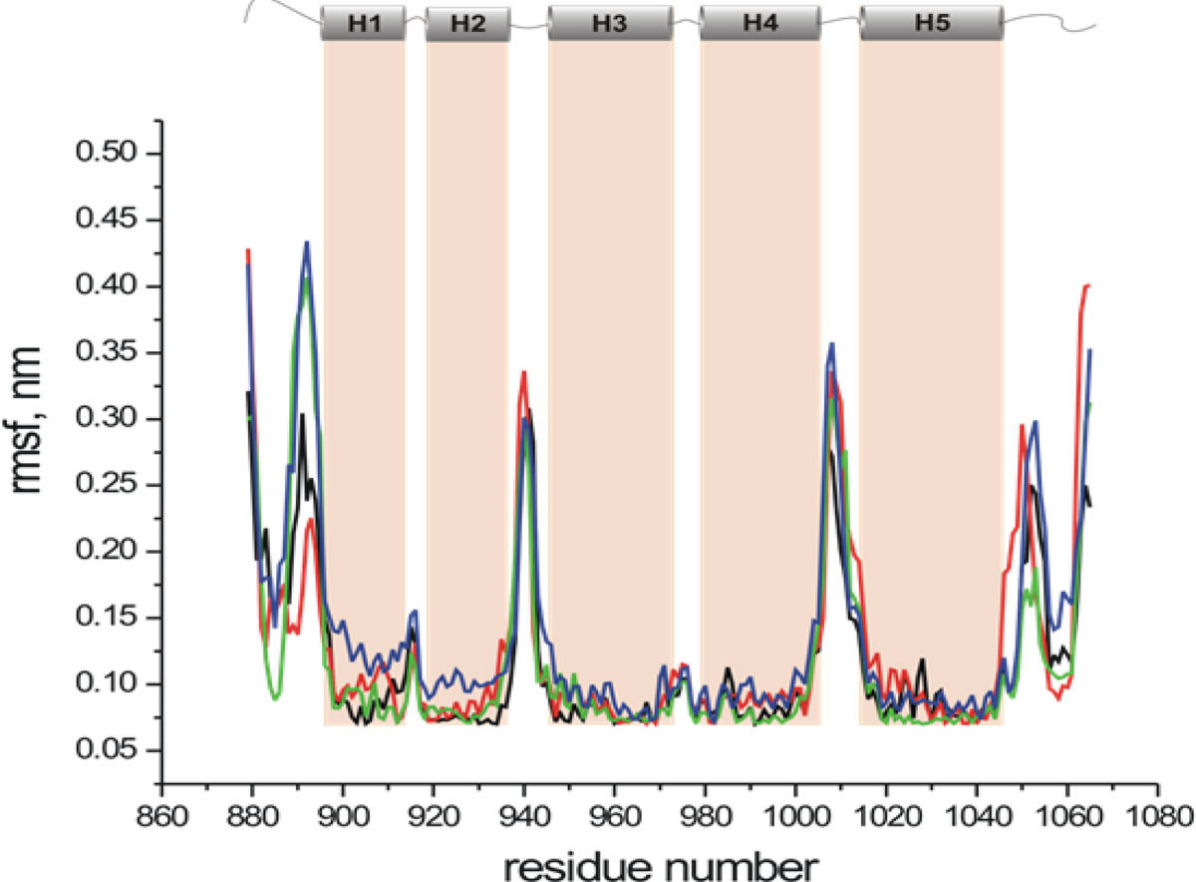
**Root mean square fluctuations of CA**. Root mean square fluctuations of CA as a function of residue number for all the simulations. Different colors represent different simulations trajectories of spin labeled vinculin tail protein. As may be seen, the pattern of fluctuations is similar in all cases, indicating that the MD results are yielding reliable information as to the dynamics of the system. The higher peaks represent the deviation in the loop regions and the lower values represent the fluctuations along the helices. The five helices of V_t _are also shown in the upper part of the figure.

The RMSF plot in Figure 8 shows that the trajectories are well equilibrated during the simulations. The helix, loops and terminal domains are clearly distinguished in the plot. It is interesting that the loops between H1-H2 and H3-H4 are not highly fluctuating compared to the other loops. The structural inspection suggests that these two loops are small in length and that the loop between H1 and H2 is masked by the long C- terminal end domain. The lower flexibility of the loop between H3 and H4 is associated with the central kink in helix H4 that brings one end of the H4 into strong tertiary and backbone contacts with helix 3 residues (see Figure 1).

The reorientational angle distribution for the spin label at position 901 (on H1 helix) *in water *and *in vacuo *simulations is presented in Figure 3. Similar broad distributions are found for this position both *in water *and *in vacuo *simulations. This spin label is located near the flexible loop region and also the head group of the side chain is exposed to the surface of the protein. The spin label at position 909(H1) shows a narrow distribution of β *in water *simulations as well as *in vacuo *simulations. This indicates that the spin label mobility at this position is restricted. Inspection of the structure indicates that this spin label is buried between two helices. The reorientational dynamics of the spin label at position 922(H2) is characterized by a broad distribution both *in water *and *in vacuo *simulations. This indicates that the spin label at this position is mobile. Though part of the spin label linker is buried, the nitroxide shows a significant mobility because the more flexible dihedrals χ4 and χ5 are not restricted (Figure 2). The spin label at position 927(H2) shows a moderate distribution of β *in water *simulations when compared to the position 922(H2). However the distribution of β in vacuo simulations indicates that the spin label at this position is highly restricted due to contacts with neighbouring helix atoms. The spin label at position 934(H2) shows restricted mobility, both *in water *and *in vacuo *simulations with narrow β distributions. This spin label side chain is surrounded by bulky residues. Considering the overall mobility pattern, the H2 helix shows restricted mobility with low β distributions.

Helix H3 carries three spin labels located at positions 950, 957 and 972. The distribution plots indicate that the mobility is moderate *in water *simulations, whereas in vacuo simulations the mobility is significantly increased. A very interesting distribution pattern is found in vacuo simulations of 972R1 that indicates two rotameric states of the spin label that are equally distributed (data not shown). This spin label is located near the loop region and oriented between two helices. However a functional study has shown a perturbed conformation of protein due to the bound spin label at position 972. Helix H4 has only one spin label at position 984 showing very narrow distribution of β *in water *simulations and a larger distribution in vacuo simulations. This spin label location is near to the kink region of the helix. The narrow distribution of β in water simulations reveals that this spin label has strong interactions during the simulations, whereas in the vacuo simulations the spin label is mobile. This indicates helical movement in the *in water *simulations that brings the spin label into strong tertiary contacts. H5 helix holds 1024R1 and 1033R1. Both MD simulations *in water *and *in vacuo *indicate that 1024R1 is very mobile when compared to 1033R1. 1024R1 is a surface exposed site. Though the spin label at 1033 is partially surface exposed, its mobility is restricted by the presence of an arginine residue located at 987 in the adjacent helix. 1062R1 is located on a C-terminus end that naturally shows a broad distribution of β in both *in water *and *in vacuo *simulations.

**Figure 9:**
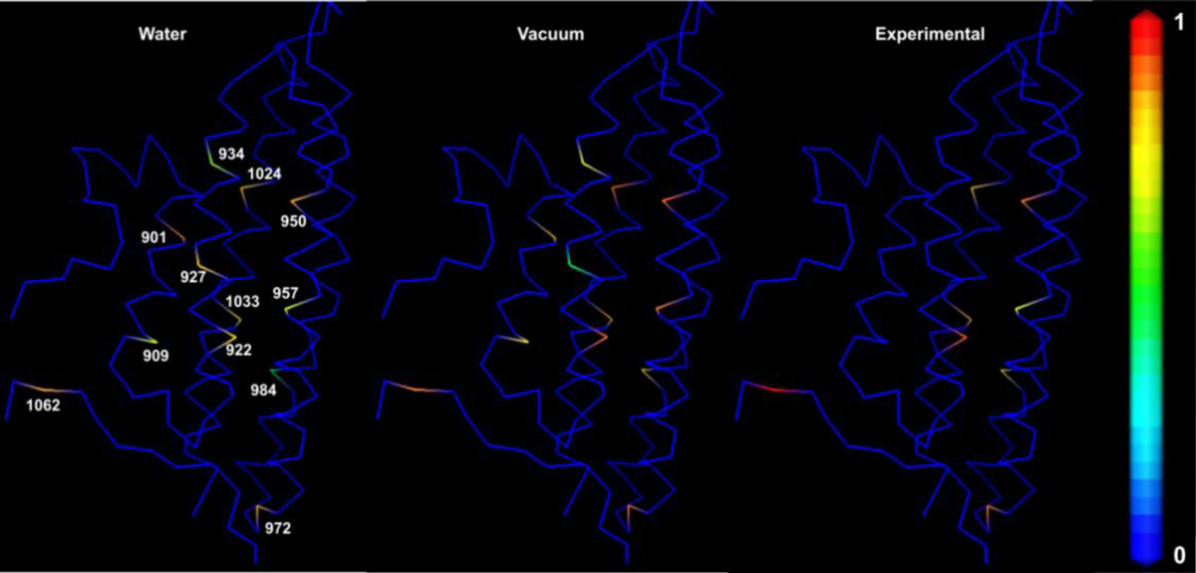
**B factor based color scheme**. Structure of vinculin tail with CA atoms represented in stick model colored by B-factor values. All the B-factor values in the coordinate file are set to zero and the mobility parameter values are replaced for the selected CA positions. Inverse line width values are used in the case of experimental values (multiplied by 1.5 times to facilitate comparison). Color coding bar is shown on the right side with the scale from zero (rigid) to one (mobile). However, experimental values for the positions 901, 909, 927 and 934 were not shown due to non availability.

As can be seen from Figures 5 and 9 the overall mobility of the spin labels calculated from *in vacuo *simulations is larger than that determined from *in water *simulations. This is most probably due to the comparably large temperature of 600 K of the vacuo simulations which allows the spin label to overcome sterical barriers which may restrict the motion at 300 K in water. One exception is position 927 where the vacuo simulations reveal a highly restricted dynamics. This might be due to the fact that the backbone motion is restricted in vacuo simulations by positions restraints of Cα and this spin label is in strong contact to a neighboring helix which cannot move. Furthermore, with this exception, the overall pattern characterized by the relative changes of the mobility is similar *in vacuo *and *in water *simulations. This pattern is also reflected in the experimental mobility parameter for the sequence 950-984 and for 1033/1062. The color coding in Figure 9 shows that the spin labels in water environments exhibit a variety of mobility behaviour depending upon the label position along the protein chains.

From Figures 5 and 6, it is clear that high mobility is associated with residues 901, 922, 950, 972, 1024 and 1062. Among these spin labels, the one at position 1062 is in the C-terminal region. 901R1 that belongs to H1 helix is immediately next to the loop region. Therefore it shows a high degree of freedom in water simulations.

The structural dynamics within a subunit of the vinculin tail protein depicted by the relative motion between spin labels 901 and 957 in helices H1 and H3 respectively is shown in Figure 7. The distance distribution shows the mean distance of 27.2 Å with an overall width of about 2 Å. The distance values between spin labels at positions 922(H2) and 957(H3) reveal a smaller distribution. A study by Palmer et al [28] suggests that conformational change in the vinculin C-terminal may depend on a critical histidine residue at position 906 in H1. The conformational change triggered by the presence of this histidine may push/pull H1 with respect to H3. The distribution of distance between 901 and 1033 is similar to the distribution between 901 and 957. It is interesting that the distance distribution between 984R1 (H4) and 1033R1 (H5) shows two distinct maxima at 7Å and 9Å respectively. It indicates that one of the spin labels fluctuates around two conformations out of which the most dominant conformation is at 7Å. This is in agreement with the observation that the angle β of 1033 shows two distinct maxima. The existence of multiple conformations in dynamic equilibrium also raises questions regarding the internal mobility of the individual side chains as well as of larger structural domains. However, the MD simulations data show that both these spin labeled positions show significant motional restriction. The simulated inter-nitroxide mean distances are in-line with the results of EPR experiments [20] (cf. Table 1).

The MD simulations data together with EPR results indicate, while the majority of the spin labeled sites in the vinculin tail show considerable dynamics, perhaps the most significant ones are those belonging to helices H1, H3, H5 and naturally the C-terminal end residue 1062. (refer to [31-33]). This dynamics might be of importance for the understanding of the unfolding process of the vinculin tail bundle and its interaction with the membrane.

## Conclusions

MD simulations were performed on the spin labeled vinculin tail domain both *in vacuo *and *in water *environment. The behavior of the spin label at various positions of the vinculin tail domain was analyzed by means of RMSF analysis and simulated EPR spectra. The results were comparable to EPR experiments. A correlation in the dynamics of the spin label mobility was found when mobility parameter values from MD simulations were compared with the EPR inverse line width data for the most of the spin-labeled sites of vinculin tail. To estimate the magnitude of helix displacements in the vinculin tail domain, distance distributions between pairs of spin label side chains were calculated and compared with experimental data. The MD simulations results in combination with EPR data show that the information contained in the spin label mobility provide a powerful means of mapping protein folds and their changes.

## Competing interests

The authors declare that they have no competing interests.

## Authors' contributions

MNV participated in design of the study, carried out the molecular dynamics simulations, data analysis, comparison of MD data with EPR data and drafted the manuscript. KPV and FDI prepared mutants and carried out the EPR experiments. ANR participated in the statistical analysis. HJS conceived of the study, participated in its design, coordination and helped to draft the manuscript. All authors read and approved the final manuscript.

## Declarations

The publication costs for this article were funded by the corresponding author's sponsor institution, DST through Ramanujan fellowship award No. SR/S2/RJN-22/2011.

This article has been published as part of *BMC Genomics *Volume 14 Supplement 2, 2013: Selected articles from ISCB-Asia 2012. The full contents of the supplement are available online at http://www.biomedcentral.com/bmcgenomics/supplements/14/S2.
